# Letter to the editor: "Fusion with and without lever reduction in degenerative lumbar spondylolisthesis—a retrospective study"

**DOI:** 10.1186/s13018-024-04537-x

**Published:** 2024-01-09

**Authors:** Kosar Fallah, Ehsan Alimohammadi

**Affiliations:** 1 Clinical Research Development Center of Imam Reza Hospital, Kermanshah University of Medical Sciences, Kermanshah, Iran; 2 Department of Neurosurgery, Kermanshah University of Medical Sciences, Kermanshah, Iran

Dear Editor,

We are writing regarding the article titled "Fusion with and without lever reduction in degenerative lumbar spondylolisthesis: a retrospective study," authored by Chao Kong, Dongfan Wang, Wei Wang, Yu Wang, and Shibao Lu, published in the Journal of Orthopedic Surgery and Research [1]. We commend the authors for conducting this study and providing valuable insights into the clinical efficacy, radiological outcomes, and complications associated with fusion surgery for degenerative lumbar spondylolisthesis (DLS).

The authors aimed to investigate the outcomes of fusion surgery with and without lever reduction in patients with DLS. Lever reduction is an innovative technique introduced to address the potential complications associated with the reduction of slipped vertebra during surgery. The study analyzed retrospective data from a registry of patients who underwent lumbar fusion surgery for DLS, with a follow-up period of at least 24 months.

The findings of this study are of significant interest to the orthopedic community. The authors observed that both the reduction group (RG) and non-reduction group (NRG) exhibited significant clinical improvement after surgery, as indicated by measures such as visual analog scale (VAS) scores for back and leg pain, Oswestry Disability Index (ODI), and the achievement of minimal clinically important difference (MCID). Importantly, there was no substantial difference between the two groups in terms of these clinical outcomes. However, the study did reveal notable differences in radiological outcomes and complications between the two groups. Patients in the RG showed statistically lower spondylolisthesis percentage (SP) and higher focal lordosis (FL) during follow-up compared to the NRG. This suggests that fusion with lever reduction has an advantage in restoring segmental spinal sagittal alignment. Additionally, the RG had a lower risk of adjacent segment degeneration (ASDeg) compared to the NRG.

The authors also reported that the overall complication rate and specific complication rates, as categorized by the modified Clavien–Dindo classification (MCDC) scheme, were similar between the RG and NRG. This finding is important, as it suggests that the introduction of the lever reduction technique does not significantly increase the risk of complications associated with fusion surgery.

Here are some important points to consider regarding the study on fusion with and without lever reduction in degenerative lumbar spondylolisthesis:

1. Study Design: The study utilized a retrospective review of prospectively collected data, which can provide valuable insights but may have inherent limitations. While a randomized controlled trial would be ideal for comparing the two surgical techniques, the authors were able to analyze a substantial number of patients with a follow-up period of at least 24 months, which strengthens the study's findings.
2. Clinical Outcomes: The study assessed clinical efficacy using self-reported measures such as the visual analog scale (VAS) for back or leg pain and the Oswestry Disability Index (ODI). These measures are commonly used in assessing pain and functional disability in patients with lumbar spine conditions. Both the reduction group (RG) and non-reduction group (NRG) demonstrated significant improvements in these measures after surgery, indicating that both techniques are effective in relieving pain and improving function.
3. Radiological Outcomes: The study evaluated radiological assessments, including spondylolisthesis percentage (SP), focal lordosis (FL), and lumbar lordosis (LL). The RG showed statistically lower SP and higher FL during follow-up compared to the NRG. This suggests that fusion with lever reduction is advantageous in restoring segmental spinal sagittal alignment. However, there were no significant differences in LL between the two groups. It is worth noting that sagittal alignment is an important consideration in the surgical management of lumbar spondylolisthesis, as it affects biomechanics and long-term outcomes.
4. Complications: The study categorized complications using the modified Clavien–Dindo classification (MCDC) scheme. The overall complication rate and specific complication rates per MCDC were similar between the RG and NRG. This indicates that the introduction of the lever reduction technique did not significantly increase the risk of complications associated with fusion surgery. However, it is worth mentioning that the overall complication rate was relatively high, highlighting the importance of careful patient selection and surgical expertise in managing degenerative lumbar spondylolisthesis.
5. Adjacent Segment Degeneration (ASDeg): The study found that patients in the RG had a lower risk of ASDeg compared to the NRG. ASDeg is a known complication following lumbar fusion surgery, and any technique that can reduce its occurrence is of clinical significance. The findings suggest that fusion with lever reduction may offer a protective effect against ASDeg, although further research is needed to confirm and understand the underlying mechanisms.

While the study provides valuable insights, it is important to acknowledge certain limitations:

1. Retrospective Design: The study utilized a retrospective design, which relies on previously collected data. This type of design is susceptible to selection bias and the potential for incomplete or missing data. Although the authors attempted to mitigate these limitations by utilizing prospectively collected data, there may still be inherent limitations associated with the retrospective nature of the study.
2. Lack of Randomization: The study did not utilize a randomized controlled trial design, which is considered the gold standard for comparing treatment interventions. The absence of randomization introduces the possibility of confounding factors and selection bias, which may influence the results. As a result, there may be inherent differences between the reduction group (RG) and non-reduction group (NRG) that could have affected the outcomes.
3. Limited Generalizability: The study focused on a specific population of patients who underwent lumbar fusion surgery for degenerative lumbar spondylolisthesis. The findings may not be generalizable to other patient populations or different types of spondylolisthesis. Additionally, the study was conducted at a specific institution, which may have unique characteristics that could limit the generalizability of the results to other settings.
4. Lack of Control Group: The study only compared outcomes between the RG and NRG, without a control group that did not undergo fusion surgery. A control group would have provided a reference point for comparing the efficacy and outcomes of the surgical techniques. Without a control group, it is challenging to determine the relative benefits and risks of fusion with and without lever reduction compared to non-surgical or alternative treatment approaches.
5. Limited Follow-up Period: Although the study had a minimum follow-up period of 24 months, the long-term outcomes and potential complications associated with fusion with and without lever reduction may not have been fully captured. Longer follow-up periods would provide a more comprehensive understanding of the durability and sustainability of the surgical outcomes.
6. Potential Confounding Factors: Despite attempts to control for confounding factors, there may still be unaccounted variables that could have influenced the outcomes. Factors such as patient comorbidities, surgeon experience, and variations in surgical techniques could impact the results but were not fully addressed in the study.
7. Sample Size and Power: The study's sample size may influence the statistical power to detect meaningful differences between the two groups. A larger sample size would have provided more robust results and increased confidence in the findings. Insufficient power may lead to type II errors, where true differences between the groups are not detected due to the study's limited ability to detect them.

In a recently published meta-analysis, the authors conducted a comprehensive meta-analysis to compare the outcomes of fusion in situ and reduction in the treatment of lumbar spondylolisthesis [2].

The results of the meta-analysis indicate that both fusion in situ and reduction techniques are associated with good clinical outcomes in the treatment of lumbar spondylolisthesis. However, the reduction group demonstrated some advantages over the fusion in situ group. Specifically, the reduction group had a significantly higher union rate, improved radiographic slippage, and shorter hospital stays compared to the fusion in situ group. These findings are important in guiding clinical decision-making and can potentially improve patient outcomes and resource utilization.

One of the notable strengths of this meta-analysis is the subgroup analysis performed for different types of spondylolisthesis (isthmic, moderate, and severe). This approach allows for a more comprehensive understanding of the outcomes based on the severity and etiology of the condition. Additionally, the authors conducted sensitivity analyses, which confirmed the robustness of the original results.

While the study provides valuable insights, there are a few aspects that warrant further consideration. First, it is important to acknowledge the limitations inherent in the included studies themselves, as the meta-analysis is dependent on the quality and design of the primary studies. Second, the authors appropriately addressed publication bias; however, it is essential to consider the potential influence of unpublished or non-indexed studies on the overall findings.

In another study, Chan et al. aimed to explore the impact of spondylolisthesis reduction on patient-reported outcomes (PROs) following decompression and fusion surgery for degenerative lumbar spondylolisthesis [3].The authors utilized the Quality Outcomes Database (QOD) to identify patients who underwent posterior lumbar fusion for spondylolisthesis with a minimum 24-month follow-up. The study investigated the correlation between Meyerding slippage reduction and PROs, including the Oswestry Disability Index (ODI), EQ-5D, Numeric Rating Scale (NRS) for back pain (NRS-BP) and leg pain (NRS-LP), and patient satisfaction. Multivariable regression models were employed to adjust for preoperative and surgical variables. The results of the study demonstrated that patients in both groups, those with slippage reduction ≥ 3 mm and those with slippage reduction < 3 mm, reported significant improvement in all primary PROs. However, there was no significant difference in PROs between patients with or without intraoperative reduction of listhesis. Furthermore, there was no correlation found between the magnitude of Meyerding slippage reduction and clinical outcomes. These findings challenge the assumption that radiographic improvements in Meyerding grade directly translate to better patient-reported outcomes.

The findings suggest that factors beyond the degree of slippage reduction may influence patient outcomes, highlighting the importance of considering other patient-specific variables and surgical factors in treatment decision-making.

While the study provides important contributions, there are a few aspects that require further discussion. First, it would be informative to explore the potential influence of other factors, such as concurrent spinal stenosis or degenerative disc disease, on the relationship between slippage reduction and PROs. Additionally, the study's retrospective nature may introduce inherent limitations, and it would be beneficial to consider prospective studies with longer follow-up periods to assess the long-term impact of slippage reduction on outcomes.

Overall, this study contributes valuable information to the existing literature on surgical techniques for degenerative lumbar spondylolisthesis. The findings support the clinical efficacy of both fusion with and without lever reduction, while highlighting the advantages of lever reduction in terms of segmental spinal sagittal alignment restoration and a reduced risk of ASDeg. These results can guide surgeons in making informed decisions when selecting the appropriate surgical approach for patients with degenerative lumbar spondylolisthesis.

## Data Availability

Not applicable.
